# Ankyrin repeat domain-encoding genes in the *w*Pip strain of *Wolbachia *from the *Culex pipiens *group

**DOI:** 10.1186/1741-7007-5-39

**Published:** 2007-09-20

**Authors:** Thomas Walker, Lisa Klasson, Mohammed Sebaihia, Mandy J Sanders, Nicholas R Thomson, Julian Parkhill, Steven P Sinkins

**Affiliations:** 1 Peter Medawar Building for Pathogen Research and Department of Zoology University of Oxford, South Parks Road, Oxford OX1 3PS, UK; 2 Pathogen Sequencing Unit, Wellcome Trust Sanger Institute Wellcome Trust Genome Campus, Hinxton, Cambridge, CB10 1SA, UK

## Abstract

**Background:**

*Wolbachia *are obligate endosymbiotic bacteria maternally transmitted through the egg cytoplasm that are responsible for several reproductive disorders in their insect hosts, such as cytoplasmic incompatibility (CI) in infected mosquitoes. Species in the *Culex pipiens *complex display an unusually high number of *Wolbachia*-induced crossing types, and based on present data, only the *w*Pip strain is present.

**Results:**

The sequencing of the *w*Pip strain of *Wolbachia *revealed the presence of 60 ankyrin repeat domain (ANK) encoding genes and expression studies of these genes were carried out in adult mosquitoes. One of these ANK genes, *pk*2, is shown to be part of an operon of three prophage-associated genes with sex-specific expression, and is present in two identical copies in the genome. Another homolog of *pk*2 is also present that is differentially expressed in different *Cx. pipiens *group strains. A further two ANK genes showed sex-specific regulation in *w*Pip-infected *Cx. pipiens *group adults.

**Conclusion:**

The high number, variability and differential expression of ANK genes in *w*Pip suggest an important role in *Wolbachia *biology, and the gene family provides both markers and promising candidates for the study of reproductive manipulation.

## Background

*Wolbachia *are obligate endosymbiotic bacteria that are maternally transmitted through the egg cytoplasm and are responsible for several reproductive disorders in arthropods, such as cytoplasmic incompatibility (CI) in infected *Culex *mosquitoes [1,2] and many other insects. Although *Wolbachia *are not found in mature sperm, they can modify developing sperm, possibly via chromatin binding proteins [3], such that when they fertilise an uninfected egg embryonic development is arrested. The reciprocal cross between infected females and uninfected males is, however, compatible; *Wolbachia*-infected females therefore produce a higher mean number of offspring than uninfected females. This unidirectional CI enables *Wolbachia *to rapidly invade uninfected populations [4], and provides a mechanism for driving anti-pathogen transgenes or a lifespan-shortening phenotype into mosquito populations [5,6]. Bidirectional CI can also occur between insect populations, usually when they are infected with different strains of *Wolbachia*.

The genome sequence of the *w*Mel strain [7], a CI-inducing *Wolbachia *strain found in *Drosophila melanogaster*, revealed an unusually high number of ankyrin repeat domain (ANK) encoding genes. Ankyrin repeats, consisting of around 33 residues, have been identified in a large number of proteins [8]. Ankyrin repeats are known to mediate protein-protein interactions in eukaryotes and are present in proteins involved in very different functions including cell cycle regulation, mitochondrial enzymes, cytoskeleton interactions, signal transduction and toxins [9]. Although ankyrin repeats are common in both eukaryotic and viral proteins they are relatively rare in bacteria and their function remains largely unknown. A protein containing ankyrin repeats in the bacterium *Ehrlichia phagocytophila *was detected in the host cytoplasm and found to be associated with chromatin suggesting a possible role in host cell gene expression [10]. ANK proteins have also been shown to mediate protein-protein interactions in cyclin-dependent kinase (CDK) inhibitors. In *Nasonia *wasps, the control of host cell cycle timing at karyogamy appears to be disrupted in CI and inhibition of CDK1 has been proposed as a possible mechanism [11,12]. Taken together this has led to the suggestion that ANK genes could play a role in *Wolbachia*-induced CI [7].

Species in the *Cx. pipiens *complex display an extremely high number of *Wolbachia*-induced crossing types between populations, with a high frequency of uni- or bidirectional incompatibilities [13-15]. Despite the complexity of crossing types, no polymorphism in the *w*Pip strain of *Wolbachia*, responsible for CI in *Cx. pipiens *mosquitoes, has been found in the nucleotide sequences of *ftsZ *[15] and 16S rRNA [16] or in the highly variable *wsp *(surface protein)gene [17]. Sequencing of the *w*Pip genome was undertaken partly in order to attempt to resolve this discrepancy. Interestingly, sequence analysis of some ANK genes found in *w*Pip revealed variation in both nucleotide sequence and predicted amino acid sequence for two prophage associated ANK genes, *pk*1 and *pk*2, between *w*Pip-infected *Cx. pipiens *colonies [17]. The *w*Au strain of *Wolbachia*, found in *Drosophila simulans*, is closely related to the *w*Mel strain but does not normally induce CI [18]. The homolog of *pk*2 in *w*Au contains a premature stop codon not present in the *w*Mel homolog, which suggests it could be a candidate gene for involvement in CI in *Drosophila *[19].

Variable expression between sexes and strains of *Cx. pipiens *was detected for the *pk*2 gene, a characteristic that might be expected for genes involved in the specific modification and rescue functions between incompatible strains. Any differential expression of ANK genes between male and female *w*Pip infected adult *Cx. pipiens *mosquitoes would suggest an important function of these genes in the interaction between *Wolbachia *and its insect host. How *Wolbachia *differentially modify sperm in males as well as rescue in females is as yet unknown, but could potentially involve variability in the expression and activity of *Wolbachia *genes in male and female insect hosts. Variable gene expression in *Wolbachia *is not thought to occur at a high rate, as only a small number of regulatory genes have been identified in the *Wolbachia *genomes sequenced to date [20]. In this study, we analysed the expression profile of all ANK genes in *w*Pip in *Cx. pipiens *adult mosquitoes.

## Results

### Number and distribution of *w*Pip ANK genes

Analysis of the *w*Pip genome revealed 60 ANK genes, which are numbered sequentially in Table 1. Several ANK proteins have predicted signal peptides and transmembrane domains. Thirteen of the *w*Pip ANK genes are contained in several chromosomally integrated prophage regions, similar in sequence to the *w*Mel WO-B prophage region [7]. The ANK genes *pk*1 and *pk*2, homologues of the *w*Mel genes WD0596 and WD0636 respectively and previously shown to vary between incompatible *Culex *strains [17], are here shown to be present in multiple identical copies in different prophage regions: *w*Pip_ANK8, *w*Pip_ANK14 and *w*Pip_ANK56 in the case of *pk*1 and *w*Pip_ANK12 and *w*Pip_ANK25 in the case of *pk*2. Two sequence variants of *pk*2 in *w*Pip from different *Cx. pipiens *group colonies have been previously described and were named a and b. The *w*Pip_ANK16 gene is also homologous to the *pk*2 genes/WD0636 in the *w*Mel strain and is present in *w*Pip in all the infected *Cx. pipiens *group colonies listed. A third pair of identical prophage-associated genes are also present, *w*Pip_ANK13 and *w*Pip_ANK26, which are homologues of WD0637. Thus in total there are 56 unique ANK genes present in the *w*Pip genome.

**Table 1:** Ankyrin repeat domain encoding genes in the *w*Pip genome

**ANK gene**	**ANK repeats**	**Gene length (bp)**	***w*Mel homolog, *w*Bm homolog**	**Additional gene information**
*w*Pip_ANK1	8	3324	WD0147	
*w*Pip_ANK2	1	675		2 transmembrane domains
*w*Pip_ANK3	2	1506	WD0754	
*w*Pip_ANK4	2	1020		2 transmembrane domains
*w*Pip_ANK5	4	1215		2 transmembrane domains, DnaJ domain
*w*Pip_ANK6	3	750		2 transmembrane domains
*w*Pip_ANK7	3	642		
*w*Pip_ANK8*#*	8	1473	WD0596	Prophage associated, 2 transmembrane domains
*w*Pip_ANK9	10	8249		
*w*Pip_ANK10	4	5913		
*w*Pip_ANK11	2	1947	WD0292	Prophage associated
*w*Pip_ANK12*	3	450	WD0636	Prophage associated
*w*Pip_ANK13^+^	3	711	WD0637	Prophage associated
*w*Pip_ANK14*#*	8	1473	WD0596	Prophage associated, 2 transmembrane domains
*w*Pip_ANK15	3	813	WD0637	Prophage associated
*w*Pip_ANK16	3	486	WD0636	Prophage associated
*w*Pip_ANK17	7	3102		1 transmembrane domain
*w*Pip_ANK18	2	1026		DnaJ domain
*w*Pip_ANK19	2	498	WD0566	1 transmembrane domain
*w*Pip_ANK20	11	2358		
*w*Pip_ANK21	4	1377		2 transmembrane domains
*w*Pip_ANK22	7	2328		
*w*Pip_ANK23	2	7863		
*w*Pip_ANK24	12	2721		
*w*Pip_ANK25*	3	450	WD0636	Prophage associated
*w*Pip_ANK26^+^	3	711	WD0637	Prophage associated
*w*Pip_ANK27	2	534	WD0566	Prophage ssociated, 1 transmembrane domain, signal petide
*w*Pip_ANK28	5	7989		
*w*Pip_ANK29	7	912	WD0766, Wbm0296	
*w*Pip_ANK30	2	726		2 transmembrane domains
*w*Pip_ANK31	8	1074		
*w*Pip_ANK32	4	546		
*w*Pip_ANK33	4	864		2 transmembrane domains
*w*Pip_ANK34	1	981	WD0441, Wbm0582	Signal peptide
*w*Pip_ANK35	4	2049	WD0438, Wbm0447	2 transmembrane domains
*w*Pip_ANK36	10	1341	WD0498/WD0499	1 transmembrane domain
*w*Pip_ANK37	3	1779		2 transmembrane domains
*w*Pip_ANK38	2	1146		
*w*Pip_ANK39	3	1119		
*w*Pip_ANK40	3	1170		
*w*Pip_ANK41	5	1182		
*w*Pip_ANK42	18	3411		
*w*Pip_ANK43	1	789	WD0191	2 transmembrane domains
*w*Pip_ANK44	3	1389		1 transmembrane domain
*w*Pip_ANK45	3	1665		2 transmembrane domains
*w*Pip_ANK46	5	861		
*w*Pip_ANK47	11	2448		Signal peptide
*w*Pip_ANK48	3	753		2 transmembrane domains
*w*Pip_ANK49	3	891		2 transmembrane domains
*w*Pip_ANK50	6	864	WD0035	
*w*Pip_ANK51	1	978		
*w*Pip_ANK52	12	1977	WD0385	
*w*Pip_ANK53	2	1302		1 transmembrane domain
*w*Pip_ANK54	6	1158		2 transmembrane domains
*w*Pip_ANK55	3	1983	WD0633	Prophage associated
*w*Pip_ANK56*#*	8	1473	WD0596	Prophage associated, 2 transmembrane domains
*w*Pip_ANK57	2	519	WD0566	Prophage associated, 1 transmembrane domain
*w*Pip_ANK58	3	3687		2 transmembrane domains, DnaJ domain
*w*Pip_ANK59	7	1152		
*w*Pip_ANK60	8	1695		

The number of ANK domains as identified by Pfam, gene length (bp), the *w*Mel and *w*Bm homologous gene where there this can be clearly determined. Symbols *# *(*pk*1), *(*pk*2) and ^+ ^denote groups of prophage-associated genes with identical sequences.

Only 15 of the 23 *w*Mel ANK genes have clear homologues in the *w*Pip genome, which might reflect the high degree of heterogeneity in this group of genes. Thus, when likely paralogous groups (three non-identical homologues of WD0566 and two each of WD0636 and WD0637) and identical copies are taken into account, 37 of the 60 identified *w*Pip ANK genes in the *w*Pip genome do not have any clear homologues in the *w*Mel genome. By way of comparison, the *w*Bm strain of *Wolbachia*, thought to be a nutritional mutualist in the filarial nematode *Brugia malayi*, encodes only five ANK proteins [21], three of which are homologous to the *w*Pip ANK encoding genes.

### ANK gene expression

Transcripts were detected for all of the ANK encoding genes. For the majority, expression in adult males and females of the Pel colony was not obviously different based on agarose gel electrophoresis of RT-PCR products. The *w*Pip_ANK57 gene showed very low expression in Pel female extracts and no detectable expression in Pel male RNA extracts. *w*Pip_ANK2 and *w*Pip_ANK49 showed low levels of expression in both Pel male and female RNA extracts. RT-PCR analysis also suggested that *w*Pip_ANK38 is highly expressed in both sexes.

### *w*Pip_ANK12 and *w*Pip_ANK25

**Figure 1:**
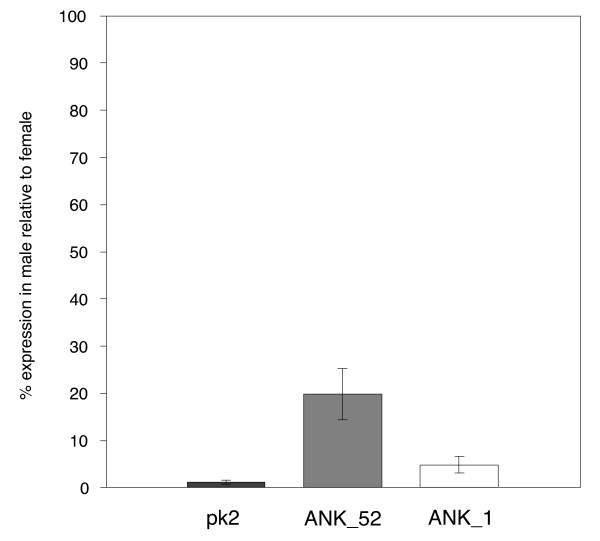
**ANK gene expression in adult *w*Pip-infected Pel males (*Cx. pipiens*)**. The mean ± SE for expression in individual adult males (6) in comparison to expression in females is shown after normalization using the *wsp *gene.

**Figure 2:**
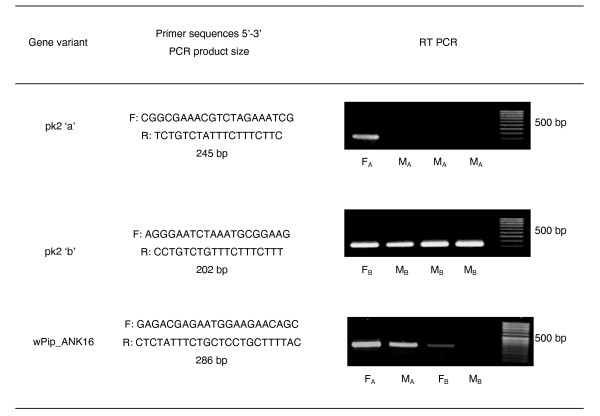
***pk*2 gene variants/homologs in *w*Pip-infected *Cx. pipiens *group colonies**. The primer sequences (5'-3') and expression levels are shown resulting from RT-PCR analysis of female (F) and male (M) RNA extracts of adult *w*Pip-infected adults.

The identical prophage associated ANK encoding genes *w*Pip_ANK12 and *w*Pip_ANK25, previously together named *pk*2 [17], showed the greatest difference in expression between sexes, with no detectable RT-PCR products in the males of the Pel and Mol colonies. Expression of these genes was also not detected in males for an additional *Cx. pipiens *colony from Sri Lanka (Sumo Cyppe). Quantification of expression by quantitative reverse transcription (qRT-PCR) was carried out and the mean male expression of the *pk*2 gene in the Pel colony in comparison to female expression was 1.6% (Figure 1). However, expression of *pk*2 was observed at similar levels in males and females of the Col colony. Primers were designed to discriminate between *pk*2 sequence variants *pk*2a present in the Pel, Sumo and Mol colonies and *pk*2b present in the Col colony and confirmed no detectable expression of *pk*2a from male RNA extracts of the Pel, Sumo Cyppe and Mol colonies using RT-PCR (Figure 2). The *pk*2b gene variant was expressed at similar levels in Col colony adult females and males. Further RT-PCR analysis showed *pk*2 gene expression in both preblastoderm embryos and pooled 4th instar larvae (sex undetermined) of the Pel colony. *pk*2 expression in pooled testes from 20 Pel males was just detectable but the RT-PCR product was very weak compared to those for *wsp *and *pk*1 (not shown).

**Figure 3:**
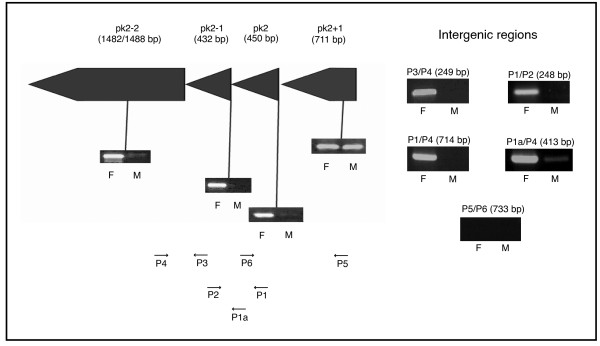
**Sex-specific expression in adult Pel colony mosquitoes for a prophage operon containing the *pk*2 gene**. The RT-PCR expression profile for male (M) and female (F) RNA extracts is shown for each gene in addition to RT-PCR products generated by using primers (P1–P6) designed to amplify fragments spanning the intergenic regions.

Differential expression between sexes was also observed for two genes directly downstream of *pk*2 (Figure 3). *pk*2-1 encodes a hypothetical protein present in identical copies in the two *pk*2 associated prophage regions. *pk*2-2 encodes a site-specific recombinase present in almost identical copies in the two prophage regions. Primers used for expression studies could not discriminate between the *pk*2-2 copies. For the gene upstream of *pk*2, also an ANK encoding gene present in two identical copies (*w*Pip_ANK13 and *w*Pip_ANK26), RT-PCR followed by agarose gel electrophoresis revealed similar expression levels in both female and male RNA extracts of the Pel colony. Primers designed to span the intergenic regions of *pk*2/*pk*2-1/*pk*2-2 produced RT-PCR products from females but no detectable products from males of the Pel colony. Primers spanning the intergenic region between *pk*2 and *pk*2+1 (primers 5 and 6) produced no amplification of a transcript from either female or male RNA extracts of the Pel colony. However, using the same primers, a product of correct size (733 bp) was amplified in both male and female DNA extracts of the Pel colony.

### *w*Pip_ANK16

The *pk*2/WD0636 homolog *w*Pip_ANK16 was present in all the *Culex *strains tested based on PCR amplification. In the Pel, Mol and Sumo Cyppe colonies, *w*Pip_ANK16 was expressed equally in males and females; however for the Col colony no expression could be detected in males, and only a weak RT-PCR product could be detected in females (Figure 2).

### *w*Pip_ANK1

Standard RT-PCR analysis followed by agarose gel electrophoresis revealed much lower expression levels of *w*Pip_ANK1 from pooled male RNA extracts of all *w*Pip-infected *Culex *colonies in comparison to female RNA extracts. Quantification of expression by qRT-PCR was undertaken and the mean male normalized expression of the *w*Pip_ANK1 gene in Pel males relative to Pel female expression was 4.7% (Figure 1). Expression levels were similar in Pel females and males for both genes flanking *w*Pip_ANK1, based on standard RT-PCR followed by agarose gel electrophoresis.

### *w*Pip_ANK52

Expression analysis using RT-PCR followed by gel electrophoresis revealed lower expression levels of *w*Pip_ANK52 from pooled male RNA extracts of all *w*Pip-infected *Cx. pipiens *colonies in comparison to female RNA extracts. Quantification of expression by qRT-PCR was undertaken and the mean male normalized expression of the *w*Pip_ANK52 gene in Pel males relative to Pel female expression was 19% (Figure 1). Reduced expression levels were also observed in Pel male RNA extracts for three additional genes flanking *w*Pip_ANK52 based on standard RT-PCR followed by agarose gel electrophoresis. However, although standard PCR using primers to span the intergenic regions of these genes resulted in an amplified product of approximately 1.5 Kb, no products were amplified using RT-PCR from either Pel female or male RNA extracts.

## Discussion

The presence of 60 ANK genes is significantly more than the 23 identified in the *w*Mel genome [7]; in fact, the number and density of ANK genes is the highest reported for any prokaryotic genome. The expansion of ANK genes in the *w*Pip strain, the degree of sequence variability and sex-specific expression in adult *Cx. pipiens *mosquitoes suggests an important biological role in parasitic strains of *Wolbachia*. The RT-PCR analysis provides strong evidence for a single transcriptional unit (operon) produced from three prophage associated genes including *pk*2. However, there was no evidence that *w*Pip_ANK1 and *w*Pip_ANK52 are part of sex-specifically regulated operons.

The quantitative RT-PCR analysis in this study represents only an estimation of differences in relative ANK gene expression. The accurate quantification of RNA expression in bacteria has been limited due to the absence of reliable standardization. In eukaryotic cells, stably expressed housekeeping genes can be used as standards to perform relative quantification of gene expression. For an endosymbiotic bacterium such as *Wolbachia*, comparing the expression of ankyrin genes to the surface protein encoding gene (*wsp*) was used to normalize for variation in *Wolbachia *density but any differences in levels of *wsp *expression between sexes and stages could be a confounding factor. The *wsp *gene was previously shown to be expressed in all *Cx. pipiens *life stages including male and female adults [22].

As some of the ANK proteins have predicted signal peptides and transmembrane domains, it is possible that they are secreted into the mosquito cytoplasm or presented on the surface of the bacterium, which could suggest that they are involved in *Wolbachia*'s interaction with the host. A proteomics analysis including experiments such as immunolocalisation studies could be used to characterize the function of ANK proteins in *Wolbachia*. Current limitations to such studies include the difficulty of obtaining epitope specificity and the absence of a transformation system for *Wolbachia*. As co-regulated genes are highly likely to show functional interactions, studies to examine the role of the co-expressed prophage-associated genes adjacent to *pk*2 are also needed.

Associations between ANK gene sequence variants and particular crossing types have previously been reported [17], enabling use of these variants as markers to further investigate *Wolbachia*-induced CI in the *Cx. pipiens *group. The significance of sex-specific expression patterns in the *pk*2 genes in some host strains but not others is not yet understood, but its occurrence did not correlate with the crossing patterns described in Table 2. The Mol and Pel colonies are bidirectionally incompatible with each other but both show the same sex-specific expression of the *pk*2 genes in adult mosquitoes. Given the complexity of the phenotype in the *Cx. pipiens *group, it seems plausible or even probable that the genetic basis for these crossing type differences involves multiple *Wolbachia *genes, and factors such as the mosquito nuclear background interacting with *Wolbachia *variants can also contribute [17]. A hypothesis that variation at just one 'CI gene' could explain all the crossing type variation observed seems increasingly unlikely. Given the rapid evolution of ANK genes, sequence differences at particular ANK loci between crossing types does not necessarily mean that there is a causal link. However the differential expression between sexes of several ANK genes (including non prophage-associated genes) in *w*Pip does provide further support for adaptations to sex-specific interactions with its host.

**Table 2:** Percentage embryo hatch in crosses between the colonies, using 50 individuals of each sex and counting hatch rates of a minimum of eight individual egg rafts

	**Pel male**	**Col male**	**Mol male**
**Pel female**	-	72.92 ± 0.96	0.17 ± 0.12
**Col female**	44.33 ± 2.39	-	0.44 ± 0.19
**Mol female**	0 ± 0	93.49 ± 1.17	-

## Conclusion

The number of ANK genes in the *w*Pip genome is the highest yet reported in a prokaryote. The sex-specificity observed in patterns of expression for some of these genes and the differential expression between mosquito strains are also very unusual features, particularly given the generally very high level of sequence conservation between *w*Pip variants. The elucidation of the functional roles and mechanisms of evolution of this family of genes will provide many insights into the biology of reproductive parasites.

## Methods

### Identification of ANK genes and primer design

Putative protein-encoding genes were identified in the *w*Pip genome using ORPHEUS [23], followed by manual curation. The translated gene sequences were searched against the Interpro database using Interproscan [24] in order to locate ankyrin repeats and other protein motifs such as signal peptides and transmembrane domains. The protein sequences containing ANK domains were compared to the protein sequences of *Wolbachia *strain *w*Mel using blastp in order to identify possible homologs. Identification of a putative origin of replication and the assignment of ANK gene numbers was based on the location of the *dnaA *gene. Gene specific primers with an annealing temperature ranging between 50–55°C were designed for all unique ANK genes (Additional file 1) using Primer Select 5.06 (DNAstar, Madison, WI, USA) and Primer3 [25]. The unfinished sequence of the *w*Pip genome and the corresponding preliminary annotation of the ANK genes are available from the Wellcome Trust Sanger Institute website, and will be updated as the sequence is completed [26].

### Mosquito colonies

Colonies of *w*Pip-infected *Cx. pipiens *mosquitoes were selected for the study. Table 3 lists the colonies used in addition to the location of where the colonies originated. All mosquito colonies were reared using standard rearing procedures at low larval densities in insectary conditions (26°C and 70% relative humidity) with a 12:12 h light/dark circadian cycle. Mass crossing experiments between *Cx. pipiens *colonies were carried out using 50 virgin individuals of each sex. The F_1 _generation progeny from the crosses was analysed by calculating the percentage of hatched embryos from a minimum of eight egg rafts, each containing between 50–110 eggs per raft, as a measure of the CI phenotype. Female spermathacae were examined for the presence of sperm if the hatch rate was low to confirm insemination.

**Table 3:** List of mosquitoes used in the study with the colony/strain in addition to the origin where the colony/strain was first obtained

**Mosquito species**	**Colony/strain**	**Origin**
*Culex quinquefasciatus*	Pel	Sri Lanka
*Culex quinquefasciatus*	Col	Colombia
*Culex molestus*	Mol	China

### PCR

All ANK gene primers were tested on Pel genomic DNA extracted using a modified version of the Livak buffer method with ethanol precipitation [27]. Genomic DNA was PCR amplified in 2.5 mM MgCl_2_, 0.25 mM dNTPs, 0.5 μM forward and reverse primers, 0.2 units of *Taq *polymerase (Sigma-Aldrich, St Louis, MO, USA), *Taq *polymerase buffer (1×) and filter-sterilised water in a total volume of 20 μL. Standard PCR cycling conditions involved denaturing the samples for 5 min at 94°C, variable annealing temperature and 72°C (1 min each), followed by a 10 min extension at 72°C using an Applied Biosystems GeneAmp PCR system 2700 (Foster City, CA, USA). PCR assays were optimised by testing at numerous annealing temperatures.

### RNA extraction

Total RNA was extracted from young (1–2 days post eclosion) adult mosquitoes using Tri Reagent (Sigma-Aldrich) followed by chloroform extraction and isopropanol precipitation. RNA extracts were treated with DNase I (Sigma-Aldrich) to remove any contaminating DNA. As the density of *Wolbachia *is significantly lower in adult male *Culex *mosquitoes compared to females, three adult *Cx. pipiens *male mosquitoes were pooled prior to RNA extraction to increase the amount of *Wolbachia *RNA present for analysis. RNA extraction of testes was carried out by dissection of 20 Pel colony males under a dissecting microscope in 0.1% saline after immobilising adult mosquitoes on ice. Dissected testes were rinsed in PBS and then pooled in 1.5 mL microcentrifuge tubes in RNAlater (Ambion, Austin, TX, USA) to prevent RNA degradation. The quality and yield of total RNA was measured using a Nanodrop ND 100 spectrophotometer.

### RT-PCR

Reverse transcription (RT) PCR analysis was performed using the Qiagen Onestep RT-PCR kit (Hilden, Germany). RNase-free water, Onestep RT-PCR buffer (1×), 400 μM dNTPs and Onestep RT-PCR enzyme mix were combined with gene specific primers (0.6 μM) to amplify 2.0 μL of template RNA in 50 μL reactions. Reverse transcription was carried out at 50°C for 30 min followed by 95°C for 15 min. Samples were PCR amplified by denaturing for 5 min at 94°C, cycled 35 times at 94°C (1 min) variable annealing temperature (1 min) and 72°C (1 min each), followed by a 10 min extension at 72°C using an Applied Biosystems GeneAmp PCR system 2700. A total of 10 μL of RT-PCR products and a 100 base-pair marker (Sigma-Aldrich) was electrophoresed on 1% agarose gels stained with ethidium bromide and visualized under ultraviolet illumination. To examine for false positives that might result from amplification of DNA, parallel reactions without adding the reverse transcriptase (*Taq *polymerase only, Sigma-Aldrich) to the reaction mixture were included.

### Quantitative RT-PCR

Quantification of gene expression was carried out using the Qiagen Onestep SYBR green RT-PCR kit and the Opticon 2 Continuous Fluorescence Detection System (Genetic Research Instrumentation, Braintree, Essex, UK). Primers were designed to amplify ANK gene fragments of less than 250 bp. Standard curves were produced using serial dilution of RNA extracted from adult female mosquitoes and relative male RNA extract expression of ANK genes measured in comparison. Quantitative RT-PCR cycling conditions were 50°C for 30 min followed by 95°C for 15 min. Samples were cycled 40 times at 94°C (15 s), 55°C (30 s) and 72°C (30 s) followed by a read step. A melting curve was constructed between 50°C and 90°C. Quantitative RT-PCR assays were carried out on six male RNA extracts in two separate assays. Comparing the concentration of cDNA amplified from ankyrin genes to the *wsp *gene was used for normalization of the data, to control for both differences in extraction efficiency and also the higher *Wolbachia *density that occurs in adult female mosquitoes compared to males. The mean relative expression levels of the *wsp *gene in Pel males, used to normalize for differential *Wolbachia *density in individual adult mosquitoes, was found to be 44.2 ± 9.6% compared to expression levels in Pel female RNA extracts.

## Competing interests

The author(s) declares that there are no competing interests.

## Authors' contributions

TW designed and conducted expression experiments and analyses, LK contributed to primer design, genome analyses and annotation, MS, MJS, NT and JP carried out genome analyses and assembly, and SPS contributed experimental design, co-ordination and analyses. TW, LK and SPS wrote the paper and all authors read and approved the manuscript.

## Supplementary Material

Additional file 1 *w*Pip ANK gene primers. ANK gene primers, optimal PCR annealing temperatures and PCR product sizes used for reverse transcription PCR analysis. Identical prophage-associated genes at different locations in the genome are listed together. Click here for file
