# Supplementary material for: Ankyrin repeat domain-encoding genes in the wPip strain of Wolbachia from the Culex pipiens group
Source: BMC Biol. 2007 Sep 20;5:39. doi: 10.1186/1741-7007-5-39 (PMC2045654; doi:10.1186/1741-7007-5-39)
Supplement: Additional file 1 — wPip ANK gene primers. ANK gene primers, optimal PCR annealing temperatures and PCR product sizes used for reverse transcription PCR analysis. Identical prophage-associated genes at different locations in the genome are listed together. [file 1741-7007-5-39-S1.doc]

**Additional data file 1. *w*Pip ANK gene primers.**

| ANK gene | Primer sequences (5’-3’) | PCR annealing temperature (°C) | PCR product size (bp) |
| --- | --- | --- | --- |
| wPip_ANK1 | F: ACGGCTCTGAAACGTTAGAAT | 50 | 229 |
|  | R: TCTTGGCCCTCCTTTTGTAA |  |  |
|  | F: GATCAAAAATGGGATTCGGTAGA | 51.5 | 610 |
|  | R: CTTCTTGGCCCTCCTTTTGT |  |  |
| wPip_ANK2 | F: ATCTTCGCTGCATATACCTTGAC | 52 | 527 |
|  | R: GCTGGAATGACACCTTACCTGA |  |  |
| wPip_ANK3 | F: TGCAAGTGCAGAGCAACCTTAC | 56 | 257 |
|  | R: CTGAGAGCGTGAATTCTTTCCTGAT |  |  |
| wPip_ANK4 | F: ATGAAAATGGGAATACACCTCTAC | 52.5 | 405 |
|  | R: CTATCCCTAACGCTAACATTCCTG |  |  |
| wPip_ANK5 | F: CAGTGGGGAATTTATTGTAGGTAGA | 51 | 771 |
|  | R: TATTTTTCGCGTTTATCGTTGTC |  |  |
| wPip_ANK6 | F: TACAACCCCCTAAACTAAAAACATT | 52 | 480 |
|  | R: TATAACCCAACATTGCACCTAACG |  |  |
| wPip_ANK7 | F: AGGGGTATATGCCATCACAAAAAC | 53.5 | 444 |
|  | R: TAGGAAGAGGCAAGCGAGGTATT |  |  |
| wPip_ANK8 | F: TATCTTCTCGCGTTAGTAAAACTTC | 53 | 520 |
| wPip_ANK14 | R: AATAAATACGGGTCAGAACAAGATA |  |  |
| wPip_ANK56 |  |  |  |
| wPip_ANK9 | F: ATGAAAGGGATGGTTGAAGAAGGAC | 53 | 713 |
|  | R: TGAGCCGCTAACCATAAGGGACTA |  |  |
| wPip_ANK10 | F: TTGCTGCGCTAAAAGGTCCATA | 52 | 542 |
|  | R: GAAAAAGCAGTTGCCGAATCC |  |  |
| wPip_ANK11 | F: GCAGGAGCAAATATTGGTGGAT | 52.5 | 400 |
|  | R: TTCCTTGCCGAATATTTAGCAC |  |  |
| wPip_ANK12 | F: TCATAAATAGCTTTTCAATTCTGTCT | 50 | 450 |
| wPip_ANK25 | R: GGTAGATTATGATAAAGCTGGTAAG |  |  |
| wPip_ANK13 | F: CTCCGGCATGTAGCTTCCATTC | 55 | 486 |
| wPip_ANK26 | R: TTACACCAAGCAGTAGAAATCTCCA |  |  |
| wPip_ANK15 | F: GATGTACGCCACTCCACTCTGC | 54 | 562 |
|  | R: ACCTTCCTTTTCGCTACCTCATCTA |  |  |
| wPip_ANK16 | F: TAAATCAGCAAAAGAATTATTAGA | 50.5 | 404 |
|  | R: ATACCTCGGTTATCTCTTTACTATT |  |  |
|  | F: GAGACGAGAATGGAAGAACAGC | 55 | 286 |
|  | R: CTCTATTTCTGCTCCTGCTTTTAC |  |  |
| wPip_ANK17 | F: GCAGGGCACAAGCTCATTAGA | 53 | 744 |
|  | R: TTCCCTGCAAGCTTAGTTTTTATTC |  |  |
| wPip_ANK18 | F: AGGGGTGTCAAGAAATGCTACTC | 51.5 | 560 |
|  | R: TACTGCCTTGCTTCGATAAAACA |  |  |
| wPip_ANK19 | F: GCTGCATTTTTGGTGGCTTAT | 52 | 439 |
|  | R: ATCGTAGGGCTTGTCTTTGTTGT |  |  |
| wPip_ANK20 | F: CCGCAAAAGGCAATAGAAAAGT | 53 | 441 |
|  | R: TTGCGCCATAACATAGTAAAGTCAT |  |  |
| wPip_ANK21 | F: CACAAAGGCAGTTAAAAGAAGAGAT | 52 | 804 |
|  | R: GTAGAGCCGTCACCAGTCAAGT |  |  |
| wPip_ANK22 | F: AAAGTGTAAATCTATGGGGGAATGA | 51 | 405 |
|  | R: TTTTTGCCTCTTTTACCTTACCAGT |  |  |
| wPip_ANK23 | F: TGTTTGCCGAGTACGAGTTCAT | 51.5 | 594 |
|  | R: TAATTCTTCTTCCGTACCGTTTCTA |  |  |
| wPip_ANK24 | F: CTTTGCACGATGCTGCTGAG | 56 | 432 |
|  | R: CTACCGCATCCAGCCTACCAT |  |  |
| wPip_ANK27 | F: ACAGTATTACATTCTGCCGC | 53 | 151 |
|  | R: AAGGTTTATCTTTGTTGTGCC |  |  |
| wPip_ANK28 | F: GGCGGCAATATAATCCTAACG | 52 | 984 |
|  | R: CAATGTCTTTTTCGAGCCTACTGA |  |  |
| wPip_ANK29 | F: CATCTTTCGAGCCCAGTCAATAG | 57 | 710 |
|  | R: TGATTTACGGAGGTGGGAAGATAGT |  |  |
| wPip_ANK30 | F: GACCTCCCTGCTTATCAAGAGTGG | 52 | 443 |
|  | R: ATCCCAGCCATAGCACCTACTGATA |  |  |
| wPip_ANK31 | F: CTTAGCCTCTCAGAACGGACATACT | 53 | 484 |
|  | R: CGAGCCAACTAACACGAAGAATC |  |  |
| wPip_ANK32 | F: CGTTGAGAGCCAAACAAGATACTAA | 52.5 | 434 |
|  | R: GATGCAGCTTGTACTTGGAGTCTC |  |  |
| wPip_ANK33 | F: GGAAAAGTACGAGGACGATTGA | 52 | 564 |
|  | R: GTGCTGCTGCTGTAACTGC |  |  |
|  | F:AAGGGTGTACTCCTTTACATTTC | 53 | 150 |
|  | R: CGTCTTGATTCTTAGCATAACC |  |  |
| wPip_ANK34 | F: AGACAATATGATAGCCTAAATGAC | 55 | 260 |
|  | R: ACTACTCTAGGATCAGCACCTTGTA |  |  |
| wPip_ANK35 | F: GAATCGCTTAGAGAACAGCGAAAAA | 51.5 | 748 |
|  | R: TGGTATGGGCTATAACTTCTTCAA |  |  |
| wPip_ANK36 | F: ACAAAATGCAACTGGTAAAGC | 53 | 153 |
|  | R: TTCCCTTTGAAACAAGAAATG |  |  |
| wPip_ANK37 | F: GCTGCTAGAGGTGGCTTTGA | 52 | 643 |
|  | R: ATTCTTTTGAGTGCGTTGCTTTAT |  |  |
| wPip_ANK38 | F: TAGCTTAGAGATAGTGCGGTTTTTG | 52 | 492 |
|  | R: TATCTTAGTATTTGCCCTGGTGGAG |  |  |
| wPip_ANK39 | F: ATAAGAGATGCGGGTGGTAGAATA | 52.5 | 606 |
|  | R: TTTGATGATGCACGCCTTTAGATA |  |  |
| wPip_ANK40 | F: ATCTTTTCTAGCATTTCCAACAAAC | 50 | 340 |
|  | R: GAAACCTATACATCATGCTACTCT |  |  |
| wPip_ANK41 | F: ACAAGAATAGTCCGGAAAAAGTG | 52.5 | 657 |
|  | R: ATGTCAAAAGCGAATTATCCAGT |  |  |
| wPip_ANK42 | F: ACTTTTCCGTTACTTGCTGCTATTA | 53 | 578 |
|  | R: TTACCGCCTTTGCTATCTATTACAT |  |  |
| wPip_ANK43 | F:TTTTAAAATATGGTGCAAGCC | 53 | 222 |
|  | R:TCATTGAAGGGACTAAAGCAC |  |  |
| wPip_ANK44 | F: ACGATGTGCCTTTTTCCAGTAG | 51.5 | 506 |
|  | R: CGGCTTTTCATTCTCTTGCTTAT |  |  |
| wPip_ANK45 | F: ATCAACAGCCGCAAAGAAACATT | 53.5 | 858 |
|  | R: GCGCAGAAGCAGCTACAACATAC |  |  |
|  | F: AAAGAAAGCTCACCATCACTG | 53 | 223 |
|  | R: GCTTTGCTCATTAATTCTTGG |  |  |
| wPip_ANK46 | F: AGTATTGTCCAAGTATGCCTGTCA | 51.5 | 465 |
|  | R: AATGTCCACTATATGCTGCTTCA |  |  |
| wPip_ANK47 | F: ATGGCCAGAGCAATTAGTGAGATAG | 51 | 536 |
|  | R: TTAGCCGCATAGTGTAGTGGTGTT |  |  |
| wPip_ANK48 | F: TTGGCACGAATTATTGAGTTTAGTT | 52.5 | 705 |
|  | R: TGTCGATTTGCTGAGATTGGTTTAT |  |  |
| wPip_ANK49 | F: CTAAAGGCAAGCAATGAAGAGGTAT | 52 | 540 |
|  | R: AATTGCTGATGTGCGTTGTTTT |  |  |
| wPip_ANK50 | F: TGATCAAGGAGATAAAAGATGAGT | 50 | 476 |
|  | R: TAAAGCTGTCCGCCCATTATT |  |  |
| wPip_ANK51 | F: TGATATGCCTCGCTTTACTGATGGT | 53.5 | 648 |
|  | R: GTGCTGGAATTTGTGGATGACTGTT |  |  |
| wPip_ANK52 | F: ATTGGGCTGTTAAAAATAATCATTC | 51.5 | 1094 |
|  | R: ATTAACGTCTGCTCCTTTATCAAGT |  |  |
|  | F: GGAGCAAAAATTGATGCAAAA | 50 | 210 |
|  | R: AACCTTTGCTCCTTTGCTGA |  |  |
| wPip_ANK53 | F: CTGCTTCCTTTAACATTTTTACTA | 50 | 403 |
|  | R: TAGAGAAGGAGAAGTTTTTGAAAT |  |  |
| wPip_ANK54 | F: AAAGAGGTATTGCGAAGGGAAGAT | 53 | 812 |
|  | R: GCAACTAAGCCACCAACAACTACAC |  |  |
| wPip_ANK55 | F: AAATACCAAAAGCTGCAAACATAGC | 56 | 588 |
|  | R: CTGCCTTCCGTAAGATCTGTGTAAT |  |  |
| wPip_ANK57 | F: ATGAGTCAACTGAAATGTAATGAAA | 50 | 409 |
|  | R: TTCCGTCGTTATCTTTTATGTTAGG |  |  |
| wPip_ANK58 | F: GGGACCCAAGAAGCCGATTAGA | 53.5 | 615 |
|  | R: AAGCGTGTTTTGCCTCCAGATTAGT |  |  |
| wPip_ANK59 | F: CTGGCCAGAGATGATAATGA | 50 | 980 |
|  | R: AATGCTTTTTGGTGTCTTTC |  |  |
| wPip_ANK60 | F: ATAGGTGTCCATAATCGCCGTAGTA | 52 | 462 |
|  | R: ACCCGTGATTTTTGCAGTAACCT |  |  |
